# Body Height, Estimated Cerebrospinal Fluid Pressure and Open-Angle Glaucoma. The Beijing Eye Study 2011

**DOI:** 10.1371/journal.pone.0086678

**Published:** 2014-01-29

**Authors:** Jost B. Jonas, Ningli Wang, Ya Xing Wang, Qi Sheng You, Xiaobin Xie, Diya Yang, Liang Xu

**Affiliations:** 1 Beijing Institute of Ophthalmology, Beijing Tongren Eye Center, Beijing Tongren Hospital, Capital Medical University, Beijing Ophthalmology and Visual Science Key Lab, Beijing, China; 2 Department of Ophthalmology, Medical Faculty Mannheim of the Ruprecht-Karls-University of Heidelberg, Germany; 3 Beijing Tongren Eye Center, Beijing Tongren Hospital, Capital Medical University, Beijing Ophthalmology and Visual Sciences Key Laboratory, Beijing, China; Zhongshan Ophthalmic Center, China

## Abstract

**Purpose:**

To examine potential associations between body height, cerebrospinal fluid pressure (CSFP), trans-lamina cribrosa pressure difference (TLCPD) and prevalence of open-angle glaucoma (OAG) in a population-based setting.

**Methods:**

The population-based Beijing Eye Study 2011 included 3468 individuals with a mean age of 64.6±9.8 years (range:50–93 years). A detailed ophthalmic examination was performed. Based on a previous study with lumbar cerebrospinal fluid pressure (CSFP) measurements, CSFP was calculated as CSFP[mmHg] = 0.44×Body Mass Index[kg/m^2^]+0.16×Diastolic Blood Pressure[mmHg]-0.18×Age[Years]-1.91

**Results:**

Data of IOP and CSFP were available for 3353 (96.7%) subjects. Taller body height was associated with higher CSFP (P<0.001; standardized correlation coefficient beta:0.13; regression coefficient B:0.29; 95% confidence interval (CI):0.25,0.33) after adjusting for male gender, urban region of habitation, higher educational level, and pulse rate. If TLCPD instead of CSFP was added, taller body height was associated with lower TLCPD (P<0.001;beta:−0.10;B:−0.20;95%CI:−0.25,−0.15). Correspondingly, higher CSFP was associated with taller body height (P = 0.003;beta:0.02;B:0.01;95%CI:0.00,0.02), after adjusting for age, gender, body mass index, pulse, systolic blood pressure, and blood concentration of cholesterol. If IOP was added to the model, higher CSFP was associated with higher IOP (P<0.001;beta:0.02;B:0.02;95%CI:0.01,0.03). TLCPD was associated with lower body height (P = 0.003;beta:−0.04;B −0.02,95%CI:−0.04,−0.01) after adjusting for age, body mass index, systolic blood pressure, pulse, blood concentrations of triglycerides, axial length, central corneal thickness, corneal curvature radius, and anterior chamber depth. Adding the prevalence of OAG to the multivariate analysis revealed, that taller body height was associated with a lower OAG prevalence (P = 0.03;beta:−0.03;B:−1.20;95%CI:−2.28,−0.12) after adjusting for educational level and gender.

**Conclusions:**

Taller body height was associated with higher CSFP and lower TLCPD (and vice versa), after adjusting for systemic and ocular parameters. Parallel to the associations between a higher prevalence of glaucoma with a lower CSFP or higher TLCPD, taller body height was associated with a lower prevalence of OAG.

## Introduction

The cerebrospinal fluid pressure (CSFP) is the counter-pressure against the intraocular pressure (IOP) across the lamina cribrosa and is part of the equation of the trans-lamina cribrosa pressure difference (TLCPD) as IOP – CSFP [1]–[4]. Previous studies have suggested that a low CSFP, or as a corollary, a high TLCPD, is associated with the pathogenesis of glaucomatous optic neuropathy, in particular in patients with so called normal-(intraocular)pressure glaucoma [3]–[7]. Up to now, the CSFP was usually only measured invasively by a direct lumbar puncture. Recent investigations showed however, that a higher CSFP is associated with younger age, higher body mass index (BMI) and higher diastolic blood pressure, so that a formula was deduced to calculate or estimate the CSFP in neurologically mostly normal subjects [8]–[10]. If a low CSFP, or correspondingly, a high TLCPD is associated with a higher prevalence of glaucoma, we postulated that systemic parameters associated with lower CSFP or higher TLCPD may also be associated with the prevalence of glaucoma. In this study we examined whether body height is related with CSFP and TLCPD and vice versa, and if yes, whether body height is additionally associated with the prevalence of open-angle glaucoma. We chose a population-based study design to avoid a potential bias due to referral-related selection of study participants. We did not assess an association with the prevalence of angle-closure glaucoma since the latter has been reported to be primarily associated with lower body stature, potentially due to the association between smaller globe size and shorter body stature [11]–[15].

## Methods

### Ethics Statement

The Medical Ethics Committee of the Beijing Tongren Hospital approved the study protocol and all participants gave informed written consent.

As described in detail previously, the Beijing Eye Study 2011 is a population-based cross-sectional study in Northern China [16], [17]. The only eligibility criterion for inclusion into the study was an age of 50+ years. Out of an eligible population of 4403 individuals, 3468 (78.8%) individuals (1963 (56.6%) women) participated with a mean age of 64.6±9.8 years (median, 64 years; range, 50–93 years). Body height and weight were measured. The blood pressure was determined with the participant sitting for at least 5 minutes under standardized conditions. The study participants had refrained from smoking and drinking of coffee, tea, or alcohol for at least 3 hours, and any exercise was not performed for the last 30 minutes prior to the blood pressure measurements. A standardized mercury sphygmomanometer was used, and the cuff size was chosen according to the measured circumference of the upper arm.

The ophthalmic examination included among other techniques tonometry, slit lamp examination of the anterior and posterior segment of the eye, biometry for measurement of the anterior corneal curvature, central corneal thickness, anterior chamber depth, lens thickness and axial length, digital photography of the cornea, lens, macula and optic disc (fundus camera Type CR6-45NM; Canon Inc, Tokyo, Japan). Using the optic disc photographs, the width of the neuroretinal rim and the diameters of the optic cup and optic disc were measured in the vertical meridian of the optic disc [18]. The vertical cup/disc diameter ratio (VCDR) was calculated. Additionally, we performed a spectral-domain optical coherence tomography (SD-OCT) for measurement of the retinal nerve fiber layer and choroidal thickness (Spectralis®, Wavelength: 870 nm; Heidelberg Engineering Co., Heidelberg, Germany) in all study participants as well as a SD-OCT for measurements of the optic nerve head (iTVue SD-OCT; Optovue Inc. Fremont, CA, U.S.A.) in a randomized subgroup of 1654 study participants. The study design and the techniques have been described in detail recently [16]–[18]. As reported previously [18], glaucoma was defined according to the optic nerve head criteria of the International Society of Geographic and Epidemiological Ophthalmology ISGEO [19], and in a second step, glaucoma was defined by a glaucomatous appearance of the optic disc. The optic nerve head was glaucomatous (1) if the inferior-superior-nasal-temporal (ISNT)-rule of the neuroretinal rim shape was not fulfilled in early glaucoma and in eyes with a normally shaped optic disc (it included a notch in the neuroretinal rim in the temporal inferior region and/or the temporal superior region); or (2) if an abnormally large cup was present in a small optic disc which normally would not show cupping. The assessment of the optic disc photographs was carried in a masked manner. Each photograph of a glaucomatous optic disc was independently adjudicated by three senior graders (LX, YXW, JBJ). The whole glaucoma group was then differentiated by findings obtained by gonioscopy or by anterior segment optical coherence tomography into subjects with open-angle glaucoma or subjects with angle-closure glaucoma.

Using the lumbar CSFP measurements obtained in a previous study, we formed a formula to estimate the CSFP [10]. That study included patients who consecutively underwent lumbar puncture for diagnosis and treatment of suspected neurological diseases. Exclusion criteria were a final diagnosis of diseases likely to be associated with a pathological CSFP, and a visual acuity lower than 20/400. The primary indications for lumbar puncture were peripheral neuropathy, intracranial hypertension, spontaneous intracranial hypotension, cavernous sinus syndrome, meningitis, multiple sclerosis, unilateral ischemic optic neuropathy, unilateral optic neuritis, optic nerve atrophy, and head injury. The study has been described in detail recently [10]. The lumbar CSFP was measured in a standardized manner at 14:00 hours. The study included 72 patients with a mean age of 42.0±13.4 years. Mean measured CSFP was 12.6±4.8 mm Hg. We randomly divided the whole group into a training group consisting of 32 patients and a test group including the remaining 42 patients, with no significant difference between both groups in age, gender, body height and weight, BMI intraocular pressure, retinal nerve fiber layer thickness, and arterial blood pressure (all *P*>0.10).

In multivariate analysis in the training group, CSFP was best described by the formula as CSFP [mmHg] = 0.44×BMI [kg/m^2^]+0.16×Diastolic Blood Pressure [mmHg] −0.18×Age [Years] −1.91. It confirmed previous investigations which had also reported on associations between higher CSFP and younger age, higher BMI and higher blood pressure [8], [9]. Applying the formula in the independent test group revealed that the measured CSFP (12.6±4.8 mm Hg) did not differ significantly (*P* = 0.29) from the calculated CSFP (13.3±3.2 mm Hg). A Durbin-Watson value of 2.08 indicated a non-significant autocorrelation for the residuals in the multiple regression models (Durbin-Watson values in the range of 1.5 to 2.5 have usually been considered to be acceptable) [20]. The intra-class correlation coefficient was 0.71, and a Bland-Altman analysis revealed that 40 out of 42 measurements were within the 95% limits of agreement. If the test group was taken as training group, the algorithm to calculate the CSFP was CSFP [mmHg] = 0.85×BMI [kg/m^2^]+0.27×Diastolic Blood Pressure [mmHg] −0.08×Age [Years] −24.8.

Inclusion criteria for the present study were availability of IOP measurements, BMI values and diastolic blood pressure measurements.

Statistical analysis was performed using a commercially available statistical software package (SPSS for Windows, version 21.0, IBM-SPSS, Chicago, IL). First, we examined the mean values (presented as mean ± standard deviation). Second, we searched in univariate analysis for associations between body height and CSFP, IOP and TLCPD and other systemic and ocular parameters. Third, we performed a multivariate analysis with body height as dependent variable and all parameters as independent variables which had a significant association with body height in the univariate analysis. We then dropped step-by-step those parameters which were no longer significantly associated with IOP, starting with the parameters with the highest *P*-values. Fourth, in a reverse manner, we checked for associations between CSFP and other systemic and ocular parameters including IOP, applying the same steps as for body height. Finally, we added the prevalence of open-angle glaucoma to the list of independent parameters to see whether the dependent variable was additionally associated with the frequency of open-angle glaucoma. All *P*-values were 2-sided and were considered statistically significant when the values were less than 0.05; 95% confidence intervals (CI) were presented.

## Results

Measurements of IOP, blood pressure and BMI were available for 6684 eyes of 3353 (96.7%) subjects with a mean age of 64.4±9.7 years (median: 63 years; range: 50 to 93 years), a mean refractive error of −0.22±2–10 diopters (median: 0.25 diopters; range: −22.0 to +7.00 diopters), and a mean axial length of 22.3±1.1 mm (median: 23.1 mm; range: 18.96–30.88 mm). The group of subjects not participating in the study as compared with the group of subjects included into the study was significantly (P<0.001) older (69.8±11.4 years versus 64.4±9.7 years), while both groups did not differ significantly in refractive error (−0.31±3.96 diopters versus −0.22±2–10 diopters; P = 0.93), axial length (23.1±1.8 mm versus 22.3±1.1 mm; P = 0.75) nor gender (P = 0.06). Glaucomatous optic neuropathy was detected in 385 (5.4%) eyes, with 256 (3.8%) eyes with open-angle glaucoma, 125 (1.9%) eyes with primary angle-closure glaucoma, and 4 (0.1%) eyes with secondary angle-closure glaucoma.

Mean body height was 161.9±8.2 cm (median: 162 cm; range: 130–180 cm). In univariate analysis, body height was associated with the systemic parameters of male gender (*P*<0.001; correlation coefficient r: −0.67), younger age (*P*<0.001; r: −0.14), urban region of habitation (*P*<0.001; r: 0.09), higher level of education (*P*<0.001; r: 0.30), lower BMI (*P*<0.001; r: −0.06), higher diastolic blood pressure (*P*<0.001; r: 0.05) and lower systolic blood pressure (*P*<0.001; r: −0.10), lower prevalence of arterial hypertension (*P*<0.001; r: −0.10), lower pulse rate (*P*<0.001; r: −0.05), higher CSFP (*P*<0.001; r: 0.06), and with the ocular parameters of more myopic refractive error (*P*<0.001; r: −0.03), longer axial length (*P*<0.001; r: 0.29), thicker central cornea (*P*<0.001; r: 0.10), anterior corneal curvature radius (*P*<0.001; r: 0.27), deeper anterior chamber (*P*<0.001; r: 0.16), thinner lens (*P* = 0.0046; r: −0.04) and lower TLCPD (*P* = 0.04; r:−0.04).

In a primary step of the multivariate analysis, body height was included as dependent variable and only the systemic parameters (which were significantly associated with body height in univariate analysis) were added as independent variables. After dropping parameters which were no longer significantly associated with body height, the model (correlation coefficient r: 0.70) eventually showed that taller body height was associated with higher CSFP (*P*<0.001) after adjusting for male gender (*P*<0.001), urban region of habitation (*P*<0.001), higher level of education (*P*<0.001), and lower pulse rate (*P*<0.001) (Table 1). The variance inflation factors of parameters in the analysis of collinearity were less than 1.7. If IOP was added to the model, IOP was not significantly associated with body height (*P* = 0.45). A similar result was obtained, if IOP corrected for its dependence on corneal thickness and anterior corneal curvature was taken (*P* = 0.93). If axial length was added, it was significantly associated (*P*<0.001; beta: 0.11; B: 0.81; 95%CI: 0.62, 1.00).

**Table 1:** Associations (multivariate analysis) between body height and systemic parameters in the Beijing Eye Study 2011 (including estimated cerebrospinal fluid pressure).

Parameter	*P*-Value	Standardized Coefficient beta	Regression Coefficient	95% Confidence Interval	Variance Inflation Factor
Estimated Cerebrospinal Fluid Pressure (mmHg)	<0.001	0.13	0.29	0.25, 0.33	1.24
Gender (Male/Female)	<0.001	−0.64	−10.5	−10.8, −10.2	1.06
Region of Habitation (Rural/Urban)	<0.001	0.06	1.00	0.64, 1.37	1.65
Level of Education (1–5)	<0.001	0.16	1.25	1.09, 1.41	1.45
Pulse	<0.001	−0.06	−0.05	−0.06, −0.03	1.00

If we took the second algorithm for the calculation of the CSFP (CSFP [mmHg] = 0.85×BMI [kg/m^2^]+0.27×Diastolic Blood Pressure [mmHg] −0.08×Age [Years]−24.8.), similar results were obtained, with body height (correlation coefficient r: 0.70) being significantly associated with CSFP (P<0.001; standardized coefficient beta: 0.06; regression coefficient B: 0.09; 95%CI: 0.05, 0.13) after adjusting for the same parameters as outlined above.

As a corollary, if TLCPD instead of CSFP was added, taller body height was associated (correlation coefficient r: 0.71) with lower corrected TLCPD (P<0.001) after adjusting for male gender (P<0.001), higher level of education (P<0.001), lower pulse rate (P<0.001), and longer axial length (P<0.001) (Table 2). The variance inflation factors of parameters in the analysis of collinearity were less than 1.5. Similar results were obtained if the dependence of the TLCPD on corneal thickness and corneal curvature were taken into account. In a similar manner, if we took the second algorithm for the calculation of the CSFP and TLCPD, similar results were obtained, with body height (correlation coefficient r: 0.70) being significantly associated with TLCPD (*P*<0.001; beta: −0.05; B: −0.07; 95%CI: −0.11, −0.03) after adjusting for the same parameters as outlined above.

**Table 2:** Associations (multivariate analysis) between body height and ocular and systemic parameters in the Beijing Eye Study 2011 (including estimated trans-lamina cribrosa pressure difference).

Parameter	*P*-Value	Standardized Coefficient beta	Regression Coefficient	95% Confidence Interval	Variance Inflation Factor
Estimated Trans-Lamina Cribrosa Pressure Difference (mm Hg)	<0.001	−0.10	−0.20	−0.25, −0.15	1.23
Gender (Male/Female)	<0.001	−0.61	−10.0	−10.5, −9.6	1.10
Level of Education (1–5)	<0.001	0.15	1.18	0.94, 1.41	1.46
Pulse	<0.001	−0.06	−0.04	−0.06, −0.02	1.00
Axial Length (mm)	<0.001	0.11	0.81	0.62, 1.01	1.15

Adding the prevalence of open-angle glaucoma to the multivariate analysis revealed, that taller body height was associated with a lower prevalence of open-angle glaucoma (*P* = 0.03; beta:−0.03; B:−1.20; 95%CI: −2.28, −0.12) after adjusting for higher level of education (*P*<0.001; beta: 0.14; B: 1.08; 95%CI: 0.87, 1.29), male gender (*P*<0.001;beta: −0.62; B: −10.0; 95%CI: −10.5, −9.6) and longer axial length (*P*<0.001;beta: 0.11; B: 0.82; 95%CI: 0.62, 1.02) (correlation coefficient r: 0.70). If the prevalence of angle-closure glaucoma instead the prevalence of open-angle glaucoma was added to the model, the association between the prevalence of angle-closure glaucoma and shorter body height was stronger (*P*<0.001; beta:−0.05; B:−2.80; 95%CI: −4.32, −1.27) than it was for the prevalence of open-angle glaucoma. As a corollary, taller body height was associated (correlation coefficient r: 0.69) with thicker retinal nerve fiber layer (as measured by confocal laser scanning tomography) (*P*<0.001; beta: 0.06; B:0.04; 95%CI: 0.02, 0.06) after adjusting for higher level of education, male gender and longer axial length (r = 0.70).

**Figure 1:**
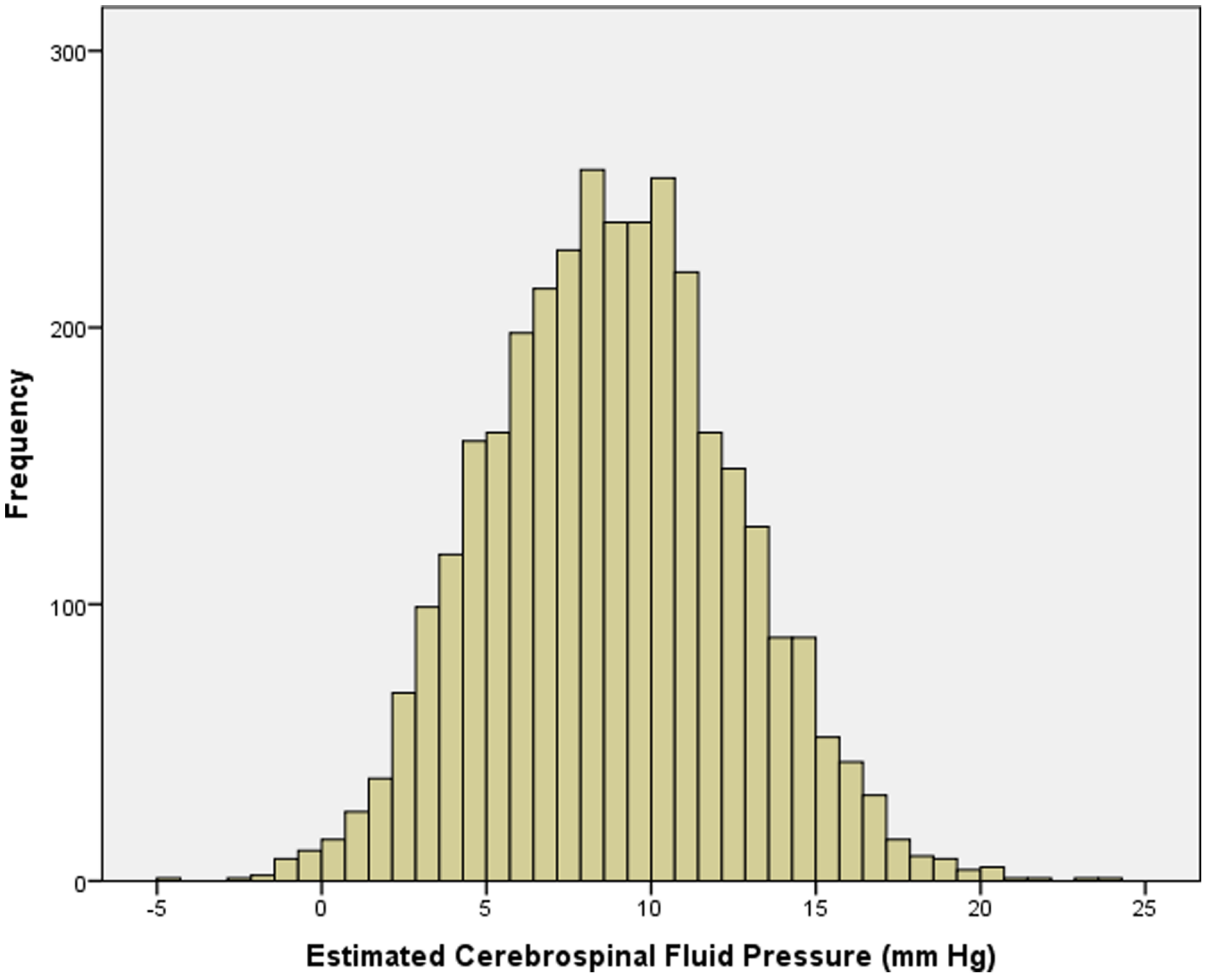
Histogram showing the distribution of the calculated cerebrospinal fluid pressure in the Beijing Eye Study 2011.

Mean estimated CSFP was 8.8±3.8 mm Hg (Fig. 1). CSFP showed a Gaussian distribution curve (*P* = 0.36; Kolmogorov-Smirnov-test). In univariate analysis, CSFP was significantly associated with younger age (*P*<0.001; r: −0.65), urban region (*P*<0.001; r: −0.42), taller body height (*P* = 0.002; r: 0.06), higher BMI (*P*<0.001; r: 0.67), higher diastolic blood pressure (*P*<0.001; r: 0.75) and systolic blood pressure (*P*<0.001; r: 0.39), higher pulse (*P*<0.001; r: 0.06), lower level of education (*P*<0.001; r: −0.19), and higher prevalence of arterial hypertension (*P*<0.001; r: 0.18), and higher blood concentration of glucose (*P* = 0.002; r: 0.07), glycosylated hemoglobin (*P* = 0.002; r: 0.04), triglycerides (*P*<0.001; r: 0.12), low-density lipoproteins (*P*<0.001; r: 0.14) and cholesterol (*P*<0.001; r: 0.08), and with the ocular parameters of refractive error (*P* = 0.02; r: 0.06), shorter axial length (*P*<0.001; r: −0.13), thinner central cornea (*P* = 0.02; r: −0.05), smaller anterior corneal curvature (*P* = 0.02; r: −0.04), thinner lens (*P*<0.001; r: −0.25) and higher intraocular pressure (*P*<0.001; r: 0.21). It was not significantly associated with gender (*P* = 0.15), blood concentration of high-density lipoproteins (*P* = 0.11), and anterior chamber depth (*P* = 0.65).

The multivariate analysis included CSFP as dependent variable and all systemic parameters which were significantly associated with CSFP in the univariate analysis. We then dropped out of the list of independent parameters those variables, which were no longer significantly associated with CSFP, starting with the parameters with the highest *P*-values. In the final model higher CSFP was associated (correlation coefficient r: 0.95) with taller body height (*P* = 0.003) after adjusting for younger age (*P*<0.001), male gender (*P*<0.001), higher BMI (*P*<0.001), higher pulse rate (*P*<0.001), higher systolic blood pressure (*P*<0.001), and higher blood concentration of cholesterol (*P* = 0.002) (Table 3). The variance variance inflation factors of parameters in the analysis of collinearity were less than 2.0. If the presence of open-angle glaucoma was added to the model, higher CSFP was associated with a lower prevalence of open-angle glaucoma (*P* = 0.009; beta: −0.01; B: −0.26; 95%CI: −0.45, −0.07). If IOP was added to the model, higher CSFP was associated with higher IOP (*P*<0.001; beta: 0.02; B: 0.02; 95%CI: 0.01, 0.03). In a similar manner, if the IOP measurements were corrected for their dependence on corneal thickness and anterior corneal curvature, higher CSFP was associated with higher values of this these corrected IOP parameter (*P* = 0.005; beta: 0.01; B: 0.02; 95%CI: 0.01, 0.04). Similar results were obtained if central corneal thickness and corneal curvature radius (which influence IOP) were added to the model. Correspondingly, higher CSFP was associated with lower TLCPD (*P*<0.001; beta: −0.20; B: −0.19; 95%CI: −0.20, −0.18) after adjusting for the same systemic parameters as outlined above.

**Table 3:** Associations (multivariate analysis) between estimated cerebrospinal fluid pressure body and body height and other systemic parameters in the Beijing Eye Study 2011.

Parameter	*P*-Value	Standardized Coefficient beta	Regression Coefficient	95% Confidence Interval	Variance Inflation Factor
Body Height (cm)	0.003	0.02	0.01	0.00, 0.02	1.98
Age (Years)	<0.001	−0.62	−0.25	−0.25, −0.24	1.17
Gender (Male/Female)	<0.001	−0.05	−0.37	−0.47, −0.28	1.95
Body Mass Index (kg/m^2^)	<0.001	0.49	0.47	0.46, 0.48	1.11
Pulse	<0.001	0.03	0.01	0.01, 0.02	1.01
Systolic Blood Pressure (mm Hg)	<0.001	0.40	0.07	0.07, 0.07	1.10
Cholesterol Blood Concentration (mmol/L)	0.002	0.01	0.05	0.02, 0.08	1.04

**Figure 2:**
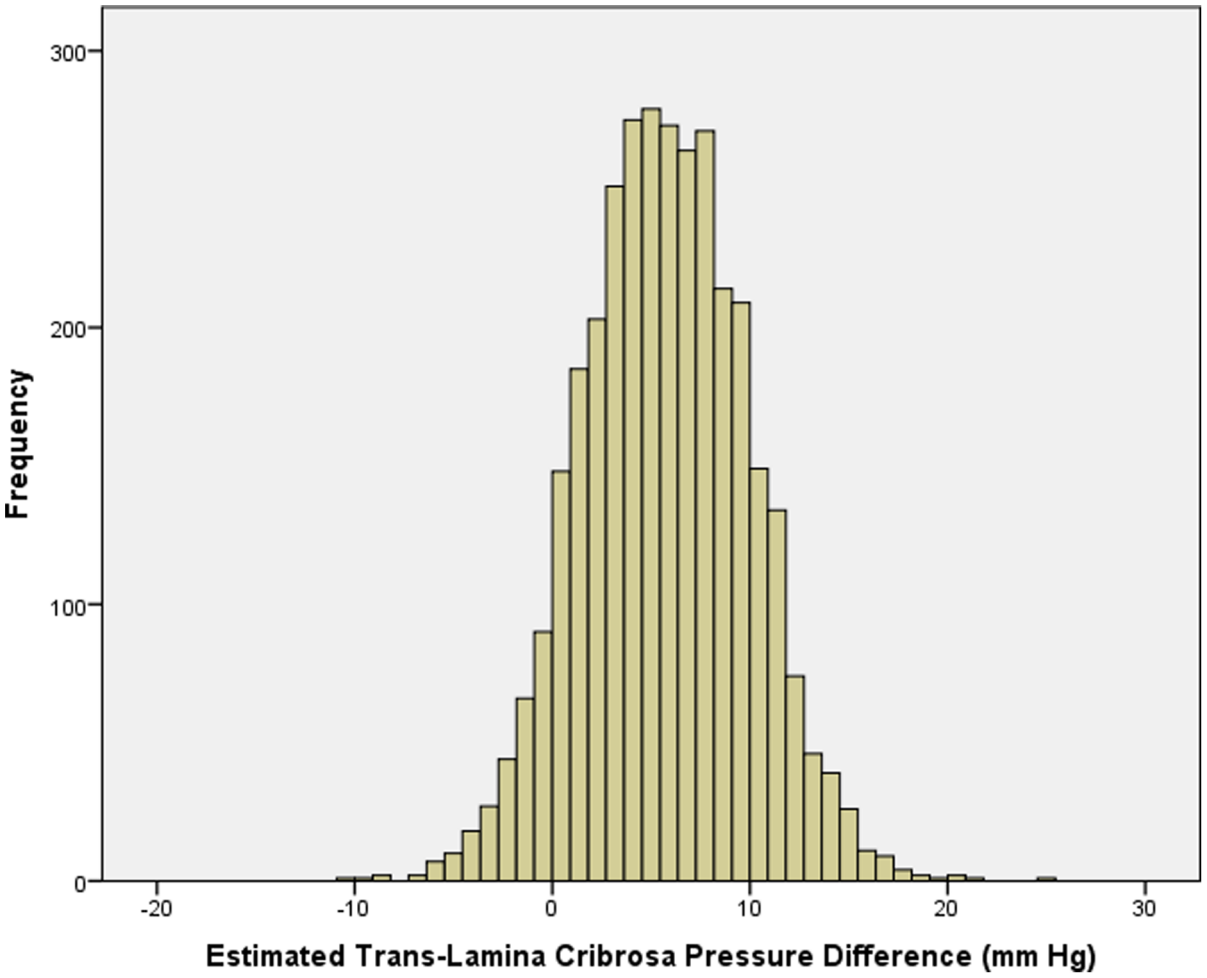
Histogram showing the distribution of the calculated trans-lamina cribrosa pressure difference in the Beijing Eye Study 2011.

Mean TLCPD was 5.9±4.2 mm Hg (Fig. 2). It showed a Gaussian distribution curve (*P* = 0.44). In univariate analysis, TLCPD was significantly associated with the systemic parameters of older age (*P*<0.001; r: 0.48), lower body height (*P* = 0.03; r: −0.04), lower BMI (*P*<0.001; r: −0.56), urban region of habitation (*P*<0.001; r: −0.33), lower diastolic blood pressure (*P*<0.001; r: −0.56), lower systolic blood pressure (*P*<0.001; r: −0.28), higher level of education (*P*<0.001; r: 0.16), and lower prevalence of arterial hypertension (*P*<0.001; r: −0.12), and lower blood concentration of triglycerides (*P* = 0.01; r: −0.05) and low-density lipoproteins (*P*<0.001; r: −0.10); and with the ocular parameters of longer axial length (*P*<0.001; r: 0.13), myopic refractive error (*P*<0.001; r: −0.09), shallower anterior chamber (*P* = 0.001; r: −0.05), thicker lens (*P*<0.001; r: 0.20), thicker central cornea (*P*<0.001; r: 0.33). It was not significantly associated with blood concentration of glucose (*P* = 0.78), cholesterol (*P* = 0.13), high-density lipoproteins (*P* = 0.14), and of glycosylated hemoglobin (*P* = 0.64), pulse (*P* = 0.81), and anterior corneal curvature (*P* = 0.21).

The multivariate analysis included TLCPD as dependent variable and all those parameters which were significantly associated with TLCPD in the univariate analysis. We dropped all independent parameters which were no longer significantly associated with TLCPD and arrived at a model, in which higher TLCPD was associated (correlation coefficient r: 0.78) with smaller body height (*P* = 0.003) after adjusting for older age (*P*<0.0013), lower BMI (*P*<0.001), lower systolic blood pressure (*P*<0.001), higher pulse (*P* = 0.03), higher blood concentrations of triglycerides (*P* = 0.03), longer axial length (*P* = 0.001), thicker central corneal thickness (*P*<0.001), smaller corneal curvature radius (*P*<0.001), and shallower anterior chamber depth (*P* = 0.006) (Table 4). The inflation factors of parameters in the analysis of collinearity were less than 1.8.

**Table 4:** Associations (multivariate analysis) between estimated trans-lamina cribrosa pressure difference, body height and other systemic and ocular parameters in the Beijing Eye Study 2011.

Parameter	*P*-Value	Standardized Coefficient beta	Regression Coefficient	95% Confidence Interval	Variance Inflation Factor
Body Height (cm)	0.003	−0.04	−0.02	−0.04, −0.01	1.20
Age (Years)	<0.001	0.41	0.19	0.17, 0.20	1.15
Body Mass Index (kg/m^2^)	<0.001	−0.43	−0.46	−0.49, −0.43	1.13
Pulse	0.03	0.03	0.01	0.00, 0.02	1.01
Systolic Blood Pressure (mm Hg)	<0.001	−0.26	−0.05	−0.06, −0.05	1.11
Triglycerides Blood Concentration (mmol/L)	0.03	0.03	0.05	0.01, 0.09	1.02
Axial Length (mm)	0.001	0.06	0.23	0.09, 0.36	1.78
Corneal Curvature Radius (mm)	<0.001	−0.08	−1.33	−1.86, 0.80	1.54
Central Corneal Thickness (µm)	<0.001	0.29	0.04	0.04, 0.04	1.03
Anterior Chamber Depth (mm)	0.006	−0.04	−0.36	−0.62, −0.10	1.21

## Discussion

In our population-based study, taller body height was associated with higher CSFD and lower TLCPD after adjusting for systemic parameters, and vice versa, higher CSFP and lower TLCPD were associated with taller body height after adjusting for systemic and ocular parameters. Correspondingly, higher prevalence of open-angle glaucoma was significantly associated with shorter body height, parallel to the association between higher prevalence of open-angle glaucoma and higher TLCPD.

Recent studies have revealed that body height is associated with ocular and general parameters. In the Singaporean Tanjong Pagar Study, body height was related to the size of eyes of adults. Taller persons were more likely to have longer globes, deeper anterior chambers, thinner lenses, and flatter corneas [13]. In the Reykjavik Eye Study, a significant relationship was found between body height and axial length, vitreous chamber depth, and radius of the corneal curvature [14]. In the Meiktila Eye Study from Myanmar, body height and weight were significantly correlated with age, gender, corneal curvature, axial length, anterior chamber depth, and vitreous chamber length [15]. In the Central India Eye and Medical Study, after controlling for age, gender, level of education, and BMI, taller subjects were more likely to have larger eyes with a longer axial length (+0.23 mm for each 10 cm increase in height), lower corneal refractive power (−0.50 diopters for each 10 cm increase in height), deeper anterior chambers (+0.03 mm for each 10 cm increase in height), and longer vitreous cavity (+0.20 mm for each 10 cm increase in height) [21]. Similar findings were obtained in the Beijing Eye Study [22]. The Beijing Eye Study 2006 also reported that patients with angle-closure glaucoma as compared to patients with open-angle glaucoma were significantly shorter in body stature in univariate analysis [11], [23]. In multivariate analysis however, the only parameter which remained to be significantly different between both glaucoma groups was anterior chamber depth. In a parallel manner, the peripheral anterior chamber depth was inversely correlated with body height in the Beijing Eye Study 2006 [24]. There were no significant associations reported between the presence of open-angle glaucoma and body stature in univariate analysis. The present analysis of the Beijing Eye Study 2011 confirms the previous study in that angle-closure glaucoma was associated with shorter body stature. The present analysis additionally suggests that also open-angle glaucoma, however to a lesser degree than angle-closure glaucoma, was associated with a shorter body stature. Our study confirms the population-based Singaporean Tanjong Pagar study, in which neuroretinal rim area was significantly and positively associated with body height [25]. In contrast, the Singapore Malay Eye Study showed in multiple regression analyses after adjustment for age, gender, optic disc size, axial length, education, family income, and intraocular pressure, that each higher body height was associated with a smaller neuroretinal rim [26].

The results of our study complement previous investigations in that a lower TLCPD and a higher CSFP may be added to the panoply of ocular and eye-related parameters which are associated with taller body stature. Taller subjects as compared to subjects with a shorter body stature had a higher CSFP and lower TLCPD. Since higher CSFP was associated with higher IOP and since TLCPD is additionally the difference of IOP – CSFP, the difference between tall subjects and short subjects was greater for TLCPD than for CSFP.

Since TLCPD is one of the main parameters for the pathophysiology of the optic nerve, in particular for glaucomatous optic neuropathy [3]–[7], the hypothesis was that if taller body height is associated with lower TLCPD, whether there is also an association between taller body stature and lower prevalence of glaucoma. In the multivariate analysis with controlling for systemic parameters of gender and educational level, taller body height was associated with a lower prevalence of open-angle glaucoma or with a thicker retinal nerve fiber layer in our study. The findings of our study thus add some hints to the assumption that besides the pressure in the intraocular space (so called intraocular pressure), the pressure in the retro-laminar compartment, i.e. the orbital CSFP, may play a role in the pathogenesis of glaucomatous optic neuropathy.

One may argue that there is a contradiction: The CSFP formula showed a higher BMI to be associated with higher CSFP; the BMI formula itself shows a higher BMI to be associated with a shorter body height; one could therefore infer that a shorter body stature should be associated with a higher CSFP. This assumption would contradict the results of our study. One may have to take into account however, that BMI was much stronger associated with higher body weight (correlation coefficient r: 0.81; *P*<0.001) than with lower body stature (r: −0.06; *P*<0.001). One of the reasons is that is that body stature enters the equation for the calculation of the BMI with its squared value. If now the association between CSFP and BMI is mainly an association between higher CSFP and higher body weight, and since taller body stature is associated with higher body weight, one may infer that taller body stature is associated with higher CSFP. Correspondingly, such an association between higher CSFP and taller body height was valid after adjusting for associations between the independent parameters in the multivariate model. It means that tall people with low BMI have a lower CSFP than similarly tall people with high BMI have. As a corollary, obese and tall people have a higher BMI than slim and tall people have, and short and thin people have a lower BMI than short and obese people. Out of all combinations, tall and obese people should have the highest CSFP, and slim and short people should have the lowest CSFP.

Potential limitations of our study should be mentioned. First, the whole statistical analysis depended on the formula to calculate the CSFP. The pilot study, in which the basis parameter for that formula were assessed included a relatively small number of subjects, and these subjects had a clinical reason to undergo lumbar puncture [10]. Although the neurological examination and the further clinical course revealed that it was unlikely that the lumbar CSFP measurement was markedly influenced by the reason to perform the lumbar puncture, one has to keep in mind, that the participants were not randomly selected normal subjects. One may also consider that the estimation of CSFP derived from a multivariate formula incorporating BMI, diastolic blood pressure, and age. The result of this formula was then termed CSFP and correlated with body height and prevalence of open-angle glaucoma, as well as with other systemic factors. Although the estimated CSFP was primarily just the result of a mathematical equation, the calculated CSFP values correlated well with invasively measured CSFP values in the independent test group in the pilot study. Nonetheless, the unknown general validity of the equation to estimate the CSFP may be most important limiting factor of our study. Second, one may argue that the statistical analysis is affected when the variable in question (here body height) is utilized in the formula used to calculate an associated variable. One may have to consider however, that body height was not a direct parameter in the equation to calculate CSFP. In addition, various multivariate analyses revealed that the association between CSFP and body height remained valid after adjusting for several systemic and ocular parameters, with the variance inflation factors for all parameters being lower than 2.0 (Tables 1–4). Third, although the associations of body height with CSFP or with TLCPD were statistically significant with *P*-values<0.05, the question arises how clinically significant they were. Fourth, lumbar CSFP measurements for the formulation of the equation to estimate CSFP were obtained in the lateral position. It has remained unclear whether and how CSFP measurements obtained in the lying position are correlated with CSFP values at the level of the brain and in the orbit in the standing position, and whether there are differences in that correlation between tall subjects and short persons. Fifth, the population of the present study included almost only Han Chinese, and it has remained unclear how body height may correlate with CFSP, TLCP and glaucoma in populations of different ethnicity. It may hold true not only for differences in the genetic background, but also for ethnic related differences in the penetrance of correlations between body height and other systemic, ocular and demographic parameters.

In conclusion, taller body height was significantly associated with higher CSFP and lower TLCPD (and vice versa), after adjusting for systemic and ocular parameters. Parallel to the reported associations between lower CSFP or higher TLCPD and higher prevalence of glaucoma, taller body height was associated with a lower prevalence of open-angle glaucoma.
